# Impact of Biochemical Parameters on Mortality in Critically Ill ICU Patients: The Role of Mechanical Ventilation, Elevated Lactate, Red Blood Cell Distribution Width (RDW-CV) Levels, and Nutrition Risk Screening 2002 (NRS-2002) Score

**DOI:** 10.7759/cureus.76522

**Published:** 2024-12-28

**Authors:** Zülküf Aras, Türkan Paşalı Kilit

**Affiliations:** 1 Internal Medicine, Kütahya University of Health Sciences, Kütahya, TUR

**Keywords:** biochemical parameters, hematological parameters, intensive care unit, mechanical ventilation, mortality

## Abstract

Objective: The mortality risk for critically ill patients in the intensive care unit (ICU) can be predicted through clinical assessments and laboratory test results. The accurate utilization of these parameters is essential for timely intervention and the initiation of appropriate therapeutic strategies. This study aims to retrospectively examine the relationship between patients’ clinical status at ICU admission, prognostic risk scoring systems, biochemical and hematological parameters, and mortality outcomes.

Materials and methods: This descriptive, cross-sectional, retrospective cohort study was conducted in the Internal Medicine Intensive Care Unit of Kütahya Health Sciences University Evliya Çelebi Training and Research Hospital, Turkey, between July 1, 2018, and July 30, 2020, and included a total of 490 patients. The initial admission data, encompassing variables such as gender, age, chronic conditions, reasons for ICU admission, ICU length of stay, total hospital stay, requirement for mechanical ventilation (MV), Nutrition Risk Screening 2002 (NRS-2002) score, hemogram, and biochemical parameters, were recorded. The clinical and demographic characteristics, along with the initial laboratory values of patients who either died or were discharged from the ICU, were subjected to statistical analysis.

Results: Of the 490 patients, 258 were male, and 232 were female, with a median age of 72 (63-80). Of the 490 patients, 211 (43.1%) died, while 279 (46.6%) were discharged from the ICU. Logistic regression analysis showed that MV requirement, NRS-2002 score, lactate, and red cell distribution width (RDW-CV) were independent predictors of mortality (p < 0.05). MV requirement had the highest odds ratio.

Conclusion: In both multivariate analysis and clinical practice, the independent predictors of mortality in ICU patients were identified as the need for MV, elevated NRS-2002 scores, increased lactate levels, and higher RDW-CV values. Among these, the strongest predictor of mortality was the requirement for MV. We anticipate that the results of our study will aid in the enhancement of mortality prediction models and provide important parameters to inform clinical practice.

## Introduction

The mortality rate in critically ill patients admitted to intensive care units (ICUs) continues to be notably high. Global studies have demonstrated that ICU mortality rates range from 14% to 41.1% [1]. With the growing demand for intensive care services, it is essential to identify key determinants influencing mortality in ICU populations and to pursue targeted research aimed at mitigating these mortality rates [2].

The mortality risk of patients in the ICU can be forecasted based on their clinical presentation and laboratory findings. Fluctuations in these parameters are instrumental in assessing the prognosis of critically ill individuals within the ICU setting. For physicians overseeing the care of ICU patients, an in-depth understanding of mortality rates and associated risk factors is imperative for implementing timely and more effective therapeutic interventions [3]. Although several predictive models, such as APACHE-II (Acute Physiology and Chronic Health Evaluation), MPM (Mortality Probability Model), and SAPS-II (Simplified Acute Physiology Score), have been developed to estimate ICU mortality, research has suggested that these models may not always provide comprehensive predictive accuracy [4].

Our study aims to explore the association between biochemical parameters and mortality in patients admitted to the Internal Medicine ICU at Kütahya Health Sciences University Evliya Çelebi Training and Research Hospital, Turkey, between July 2018 and July 2020, with the goal of contributing to the refinement of existing mortality prediction models.

## Materials and methods

The study is a descriptive, cross-sectional, retrospective cohort analysis conducted to investigate the relationship between biochemical parameters and mortality in patients hospitalized in the ICU. This study was conducted in the Internal Medicine ICU of Kütahya Health Sciences University Evliya Çelebi Training and Research Hospital between July 1, 2018, and July 30, 2020. The study included all patients aged 18 years and older who were admitted to the ICU for any reason, provided their biochemical parameters were measured upon admission. Exclusion criteria comprised patients admitted postoperatively by surgical departments for follow-up. The following patient data were systematically documented: gender, age, presence of chronic conditions, admission diagnoses, ICU length of stay, total hospital length of stay, requirement for MV, duration of MV, Nutrition Risk Screening 2002 score (NRS-2002), APACHE II score, SAPS II score, and laboratory results (including complete blood count, urea, creatinine, alanine aminotransferase (ALT), aspartate transaminase (AST), albumin, sodium, potassium, calcium, uric acid, CRP, and lactate levels). All data were retrieved from the digital records of Kütahya Evliya Çelebi Education and Research Hospital.

Ethical approval for our study was obtained from the Non-Interventional Clinical Research Ethics Committee of Kütahya Health Sciences University on August 17, 2022, with decision number 2022/08-23. The study adhered strictly to the latest version of the Declaration of Helsinki and the Good Clinical Practice Guidelines.

The study data were processed using IBM SPSS Statistics for Windows, version 23.0 (released 2014, IBM Corp., Armonk, NY) and MedCalc version 23.110 software (MedCalc Software bv, Ostend, Belgium; https://www.medcalc.org). Continuous variables were reported as medians along with interquartile ranges (IQRs), while categorical variables were represented as percentages. The Mann-Whitney U test was applied to compare continuous variables between two independent groups, and the Pearson chi-square test was used for categorical comparisons. The Kolmogorov-Smirnov test was employed to assess the normality of the data. Logistic regression analysis was conducted to determine independent predictors of mortality in ICU patients. All statistical analyses were two-tailed, with an alpha value of 0.05 set as the threshold for significance.

## Results

A total of 490 patients who were admitted to the internal medicine ICU between July 2018 and July 2020 and met the inclusion criteria were retrospectively analyzed. The demographic characteristics of these patients are summarized in Table 1.

**Table 1:** Demographic characteristics of the study patients IQR: interquartile ratio; MV: mechanical ventilation; NPPV: non-invasive positive pressure ventilation; COPD: chronic obstructive pulmonary disease; NRS: nutrition risk screening

Variable	N (%)
Age (median-IQR)	72 (63-80)
Gender	
Male	258 (52.7)
Female	232 (47.3)
MV requirement	235 (48)
NPPV	153 (31.2)
Reason for admission	
Respiratory failure	276 (56.4)
Sepsis/septic shock	167 (34.1)
Infection	337 (68.8)
Acute renal failure	255 (52.1)
Cardiovascular system	70 (14.3)
Neurological	57 (11.7)
Pneumonia	211 (43.1)
Malignancy	87 (17.8)
Diabetes mellitus	169 (34.5)
Hypertension	200 (40.8)
Chronic renal failure	101 (20.6)
Respiratory system diseases (COPD, asthma)	137 (28)
Coronary artery disease	84 (17.1)
Congestive heart failure	86 (17.6)
APACHE II score (median-IQR)	24 (18-32.3)
SAPS II score	50 (35-69)
NRS-2002 score	4 (1-6)
Length of stay in the ICU (days) (median-IQR)	4 (2-8)
Duration of mechanical ventilation (days) (median-IQR)	1 (0-5)
Days of NIMV (Median-IQR)	0 (0-2)
Death	211 (43.1)

The median age of the patients was 72 (IQR: 63-80). Of the patients, 258 (52.7%) were male, and 232 (47.3%) were female. Of the 490 patients examined, 211 (43.1%) died, while the remaining 279 (46.6%) were discharged from the ICU. The median length of stay in the ICU was four days (IQR: 2-8). MV was required in 235 patients (48%), and 153 patients (31.2%) required non-invasive positive pressure ventilation (NPPV). The median duration of MV was one day (IQR: 1-5), while the median duration for NPPV was 0 days (IQR: 0-2) (Table 1).

When the reasons for ICU admission were examined, 56.4% (n = 276) had respiratory failure, 52.1% (n = 255) had acute kidney failure, 43.1% (n = 211) had pneumonia, 34.1% (n = 167) had sepsis/septic shock, 14.3% (n = 70) had cardiovascular diseases, and 11.7% (n = 57) had neurological diseases. Infection was present in 68.8% (n = 337) of the patients (Table 1).

When evaluating chronic diseases, 40.8% (n = 200) had hypertension, 34.5% (n = 169) had diabetes mellitus, 28% (n = 137) had respiratory diseases (COPD, asthma), 20.6% (n = 101) had chronic kidney disease, 17.6% (n = 86) had congestive heart failure, 17.1% (n = 84) had coronary artery disease, and 17.8% (n = 87) had malignancies (Table 1).

When the prognostic scoring systems used for mortality prediction were examined, the median APACHE II score was 24 (IQR: 18-32.3), and the median SAPS II score was 50 (IQR: 35-69). The median NRS-2002 score used to assess nutritional status was 4 (IQR: 1-6).

When comparing the demographic characteristics of patients who were discharged from the ICU to those who died, significant statistical differences were observed in age, APACHE II score, SAPS II score, duration of MV, NRS-2002 score, and ICU length of stay (p < 0.05). Both ICU length of stay and duration of MV were notably longer in patients who died compared to those who were discharged. In addition, patients who did not survive had significantly higher age, APACHE II, SAPS II, and NRS-2002 scores than those who survived (Table 2).

**Table 2:** Comparison of patients discharged from intensive care and deceased in terms of demographic data and laboratory parameters. IQR: interquartile ratio; NPPV: non-invasive positive pressure ventilation; MV: mechanical ventilation

Variable	Discharge from intensive care (Median-IQR)	Death (Median-IQR)	P-value
Age	71 (60-79)	74 (64-81)	0.022
APACHE II score	20 (14-24)	33 (29-37)	0.00
SAPS II score	39 (30-55)	64 (47-82)	0.00
NRS-2002 score	3 (1-4)	5 (4-6)	0.00
Length of stay in the ICU (days)	4 (2-6)	5 (2-13.8)	0.01
MV number of days	0 (0-0)	3 (1-7)	0.00
NPPV number of days	0 (0-1.75)	0 (0-2)	0.34

A logistic regression analysis conducted using all parameters that were significant in univariate analysis did not yield a significant model. Therefore, a logistic regression analysis was repeated using parameters clinically associated with mortality, including age, APACHE II score, NRS-2002 score, albumin, ALT, AST, creatinine, lactate, CRP, calcium, magnesium, uric acid, leukocytes, Red blood cell distribution width (RDW-CV), MV need, respiratory failure, sepsis/septic shock, acute kidney failure, pneumonia, malignancy, and congestive heart failure. However, no statistically significant variable was identified.

Since the APACHE II score is a multivariable model that incorporates most of the parameters in our study, it may have prevented other variables from being significant. Therefore, the logistic regression analysis was repeated without the APACHE II score. In the new analysis, MV need, NRS-2002 score, ALT, AST, lactate, and RDW-CV were identified as significant independent predictors of mortality (p < 0.05) (Table 3). However, a statistically significant parameter does not always imply clinical significance. Although ALT and AST were statistically significant, their odds ratios were not clinically significant. The best odds ratio was for MV need. MV was required in 97.2% of deceased patients and was significantly higher compared to those discharged.

**Table 3:** Logistic regression analysis performed to determine the independent variables that are effective in predicting patients who died in intensive care. MV: mechanical ventilation; NRS: nutrition risk screening; ALT: alanine transaminase; AST: aspartate aminotransferase; RDW: red cell distribution width

Variable	Odds ratio	95% confidence interval	P-value
MV requirement	199	56-707	0.00
NRS-2002 score	1.3	1.07-1.6	0.01
ALT	0.99	0.98-0.99	0.02
AST	1.003	1.0006-1.0067	0.02
Lactate	1.32	1.06-.7	0.02
RDW-CV	0.95	0.92-0.99	0.01

## Discussion

The mortality rate for patients admitted to ICUs continues to be notably high, with various risk factors affecting patient outcomes widely documented in the literature. Given the rising demand for intensive care services, identifying key determinants of mortality in ICU populations and conducting research aimed at reducing ICU mortality are of paramount importance. This study sought to investigate the relationship between clinical outcomes, as well as biochemical and hematological parameters at the time of ICU admission, and the resulting mortality.

Plasma lactate levels are a widely utilized and readily accessible biomarker indicative of tissue hypoxia. Vincent et al. demonstrated that the most reliable predictor of prognosis following resuscitation in shock patients is the reduction in lactate levels within the first hour [5]. Similarly, in a study by Jan Bakker et al. on patients with septic shock, serial lactate measurements were identified as a robust predictor of multiple organ failure and mortality [6]. This study further highlighted that the duration of lactic acidosis holds greater prognostic significance than the initial lactate value. The findings from these studies align with our results, which suggest a strong correlation between elevated lactate levels and mortality in ICU patients. However, a limitation of our study is the lack of serial lactate measurements, underscoring the need for future research incorporating serial lactate assessments.

In our study, RDW-CV levels were found to be significantly elevated in patients who died compared to those who were discharged. Multivariable analysis revealed that high RDW-CV was an independent predictor of mortality. A retrospective study by Meynaar et al. on 2915 ICU patients supports our findings, reporting that high RDW at admission was associated with hospital mortality. Furthermore, RDW, APACHE II score, and age were identified as independent risk factors for mortality in the multivariable analysis [7].

The results of our study showed that NRS-2002 scores were elevated in ICU patients who died compared to those who survived. In addition, logistic regression analysis identified the NRS-2002 score as an independent risk factor for predicting mortality. Therefore, it was observed that patients with a higher degree of malnutrition had a higher mortality rate. Some studies investigating the relationship between the NRS-2002 score and mortality in ICU patients suggest that this nutritional risk assessment tool is an important prognostic indicator for critically ill patients [8,9]. These studies reported that higher NRS-2002 scores increase mortality rates. In a study by Thomas et al. examining the nutritional status of critically ill COVID-19 patients, various nutritional scales were compared, and the NRS-2002 score was identified as an independent risk factor for hospital length of stay, ICU length of stay, and mortality. In addition, it was determined that for every one-point increase in the NRS-2002 score, mortality increased by 1.23 times [10]. These studies in the literature are consistent with our study. Malnutrition negatively affects the immune system, the body’s ability to adapt and recover, and many metabolic processes. It also increases susceptibility to infections, particularly respiratory infections, leading to increased mortality [11].

This study demonstrated that mortality risk significantly increases in patients requiring MV and those with longer MV days. Multivariable analysis revealed that MV need was a significant independent predictor of mortality. A study by Ünal et al. aligns with our findings. In their research involving 111 ICU patients aged 18 and older, 44.1% required MV for an average duration of 12.7 ± 13.5 days, with an ICU mortality rate of 85.7% among those requiring MV [12]. A retrospective study by Uysal et al. involving 1033 ICU patients found that 52% of the patients required MV, with an average duration of 9.9 ± 17.6 days, and a mortality rate of 74% [13]. Studies on the impact of MV need on mortality are consistent with our findings. The high number of patients admitted to our ICU with respiratory failure, the negative prognosis associated with chronic diseases, and the provision of intensive care support to critically ill patients without resuscitation indications may explain the high mortality rates in patients requiring MV.

There are several limitations to our study. First, as this is a retrospective study, it was observed that some data were incomplete during the file review. Second, our study was conducted at a single-center, tertiary ICU with 490 patients, which is a relatively small sample size compared to other mortality studies, limiting the generalizability of the results. Third, while biochemical and blood gas parameters were recorded at the time of admission, parameters and vital signs from subsequent days were not recorded.

## Conclusions

In summary, when considering the initial admission values of critically ill patients admitted to the ICU, the most significant independent variables for predicting mortality, both in multivariable analysis and clinically, are MV needs, high NRS-2002 scores, elevated lactate levels, and high RDW-CV levels. The best predictor of mortality was the need for MV.

We believe that the data obtained from our study will contribute to the development of mortality prediction models and provide useful guidance in clinical practice.
